# Grounding Context in Embodied Cognitive Robotics

**DOI:** 10.3389/fnbot.2022.843108

**Published:** 2022-06-15

**Authors:** Diana Valenzo, Alejandra Ciria, Guido Schillaci, Bruno Lara

**Affiliations:** ^1^Laboratorio de Robótica Cognitiva, Centro de Investigación en Ciencias, Universidad Autónoma del Estado de Morelos, Cuernavaca, Mexico; ^2^Facultad de Psicología, Universidad Nacional Autónoma de México, Mexico City, Mexico; ^3^Independent Researcher, Florence, Italy

**Keywords:** context, behavioral flexibility, task selection, prediction error, cognitive robotics

## Abstract

Biological agents are context-dependent systems that exhibit behavioral flexibility. The internal and external information agents process, their actions, and emotions are all grounded in the context within which they are situated. However, in the field of cognitive robotics, the concept of context is far from being clear with most studies making little to no reference to it. The aim of this paper is to provide an interpretation of the notion of context and its core elements based on different studies in natural agents, and how these core contextual elements have been modeled in cognitive robotics, to introduce a new hypothesis about the interactions between these contextual elements. Here, global context is categorized as agent-related, environmental, and task-related context. The interaction of their core elements, allows agents to first select self-relevant tasks depending on their current needs, or for learning and mastering their environment through exploration. Second, to perform a task and continuously monitor its performance. Third, to abandon a task in case its execution is not going as expected. Here, the monitoring of prediction error, the difference between sensorimotor predictions and incoming sensory information, is at the core of behavioral flexibility during situated action cycles. Additionally, monitoring prediction error dynamics and its comparison with the expected reduction rate should indicate the agent its overall performance on executing the task. Sensitivity to performance evokes emotions that function as the driving element for autonomous behavior which, at the same time, depends on the processing of the interacting core elements. Taking all these into account, an interactionist model of contexts and their core elements is proposed. The model is embodied, affective, and situated, by means of the processing of the agent-related and environmental core contextual elements. Additionally, it is grounded in the processing of the task-related context and the associated situated action cycles during task execution. Finally, the model proposed here aims to guide how artificial agents should process the core contextual elements of the agent-related and environmental context to give rise to the task-related context, allowing agents to autonomously select a task, its planning, execution, and monitoring for behavioral flexibility.

## 1. Introduction

Cognitive robotics (CR) aims to understand cognition by recreating it in artificial agents (Asada et al., 2001; Krichmar, 2012; Cangelosi and Schlesinger, 2015; Lara et al., 2018). In doing so, the interaction with the environment is assumed to be crucial for the emergence of cognitive abilities (Pezzulo et al., 2011, 2013; Cangelosi et al., 2015). Artificial agents are considered as useful tools to explore embodied, embedded, and grounded models of cognition (Pfeifer and Scheier, 2001; Lungarella et al., 2003; Pfeifer, 2004). Here, grounded cognition is understood as a general approach that incorporates embodied, embedded, enactive, and extended cognition into a broader perspective: “cognition, affect, and behavior emerge from the body being embedded in environments that extend cognition, as agents enact situated action reflecting their current cognitive and affective states" (Barsalou, 2020b, p.2).

Artificial agents are able to explore and manipulate objects in their environments (Min et al., 2016; Adnan Mohsin Abdulazeez, 2021). However, these tasks are usually learned under controlled conditions, which restricts their ability to efficiently adapt to the demands of dynamic environments (Min et al., 2016). One of the great challenges in Cognitive Robotics (CR) is to design autonomous artificial agents that generate appropriate behaviors according to the environment in which they are situated (Mohan et al., 2013; Asada, 2020). A promising approach is the attempt to understand the underlying mechanisms of behavioral flexibility that biological agents naturally exhibit. Behavioral flexibility refers to the ability to switch from one behavior to another so as to efficiently adapt to dynamic environments (Ragozzino, 2007; Lea et al., 2020). In this regard, context processing plays an essential role in behavioral flexibility.

The processing of the current context is fundamental for biological agents to select the appropriate task at a given moment. It is widely accepted that context acts as a set of constraints that influence behavior (Bazire and Brézillon, 2005). Actually, it makes no sense to talk about appropriate behaviors without the notion of context (Turner, 1998). Furthermore, contextual information is also essential for planning the sensorimotor sequences to execute a selected task (Rosenbaum et al., 2014). It has been suggested that the brain is a context-dependent system since all inputs it processes concern the context in which they occur (Nikolić, 2010). Following this line, processing context would allow artificial agents to autonomously and appropriately prioritize goals, select appropriate tasks, plan and execute them, and even change tasks according to the current situation, ultimately showing greater behavioral flexibility.

**Figure 1:**
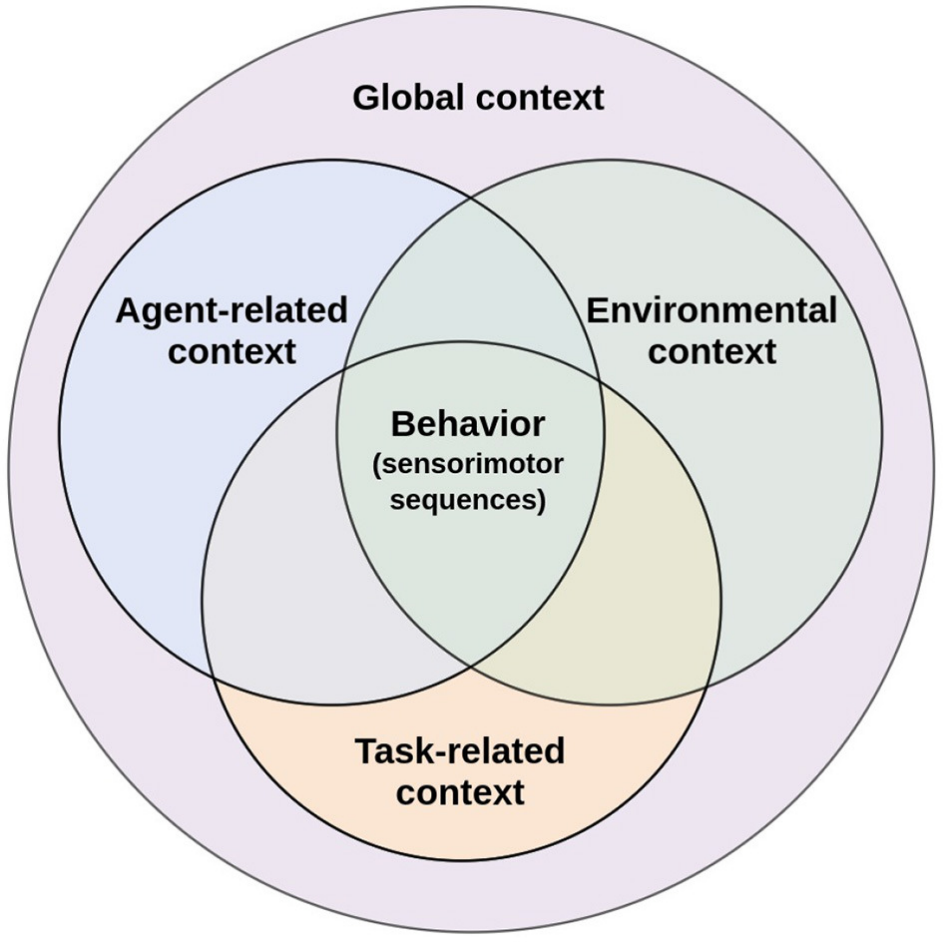
Agent-related context, environmental context, and task-related context are intertwined together to influence behavior. Figure adapted from Cohen (1995).

This paper aims to analyze the role of context in behavioral flexibility and how this concept has been used in CR. Although context is a widely used concept, not only in CR but also within cognitive sciences in general, it remains an ill-defined concept (for an attempt to analyze different definitions of the concept of context see Bazire and Brézillon, 2005). Inspired by the pioneering work of Turner (1998) in context-mediated behavior for artificial agents, here, context is defined as any identifiable configuration of environmental, task-related, and agent-related elements that are perceived and experienced as relevant in a specific moment and in a particular situation. To respond to changing conditions, biological agents must monitor internal demands and environmental factors, those that are of self-relevance and full of affect, to guide and initiate behavior (Barsalou, 2020b). Together, all those internal and external elements of a situation that have predictive power and impacts behavior constitute the global context (Turner, 1998, p.308). In order to unravel the diffuse notion of context and considering the key constituents of the definition proposed by Turner (1998), three components of the global context are considered in our analysis: agent-related, environmental, and task-related context (Figure 1). Pfeifer and Bongard (2006) considered the same components within their set of design principles for artificial agents, stating that an intelligent agent should have a defined ecological niche, a defined task, and an agent design (Krichmar, 2012).

Each type of context is constituted by a set of diverse and complex elements, and the processing of all of them in artificial agents is not computationally trivial (Brooks and Mataric, 1993; Connell and Mahadevan, 1993). In this sense, this work does not pretend to be an exhaustive study of context as such. Rather, it pretends to identify and analyzed the core elements of the agent-related, environmental and task-related context to explore how they have been taken into account in CR, and then highlight the importance of the core elements interaction for behavioral flexibility under a proposed model. Here, it is suggested that, although there are innumerable elements related to the agent, the environment, and the task, the particularity of a context is constituted by means of the specific physiological needs, motivations and associated emotions that are experienced, the perceived possibilities of action that a specific environment offers the agent, and the task configuration in a concrete environment.

An essential aspect of the proposed model is that it considers the monitoring of prediction error dynamics, which seems crucial for switching strategies under changing circumstances. One challenge for grounded cognition is to understand cognition in depth within the context of situated action cycles (Barsalou, 2020b). We suggest that through the monitoring of the core contextual elements, together with the monitoring of prediction error dynamics, artificial agents would autonomously select self-relevant situated tasks. We are aware that the sociocultural context plays an essential role in behavioral flexibility of social agents. However, we believe that it is essential to establish some core elements of the context associated with auto-regulation and object interaction before tackling more complex components of situated action cycles. In this way, artificial agents would enact situated action reflecting their current core context.

The structure of the paper is as follows: in Section 2, the role of the agent-related, environmental, and task-related context for behavioral flexibility is briefly explored and an overview of the processing of each one in biological agents is presented. In Sections 3–5, each type of context is addressed in more detail through their core elements and how these have been described in biological agents and then, some representative cognitive robotics implementations addressing similar elements are reviewed. In Section 6, the interaction of the three types of context in behavioral flexibility is explored through a schematic model that intertwines the core elements from each of them. Finally, Section 7 concludes the paper. For the remainder of the paper, when it reads “biological agents” it refers to living organisms, “artificial agents” refers to situated artificial robots and implementations and, when it reads “agents” it refers to both.

## 2. Behavioral Flexibility Through the Lens of Different Types of Context

Global context includes all internal and external elements that impact and restrict the behavior of biological agents at a given moment, enticing these agents toward the performance of certain tasks or avoiding others at any given moment. Although there are countless contextual elements, they all come from three main sources: the state of the agent, the environmental conditions, and the characteristics of the task agents are engaged with in the current moment (Cohen, 1995). This allows to identify three particular types of context: agent-related, environmental, and task-related context. This section explores the role of each type of context for behavioral flexibility in biological agents. Furthermore, how each type of context is processed by the available sensory systems of these agents will be addressed. This makes it possible to establish a basis to study the notion of context within cognitive robotics in the following sections.

Flexible behavior, the ability to select the appropriate task or change strategies to adapt to the environment, is modulated by elements associated with the biological agent and the environment (Palmer et al., 2014). The elements associated with the agent that impact behavior constitute the agent-related context, which is characterized by elements such as physiological needs, emotions, as well as postural and morphological aspects. On the other hand, the environmental context relates to the characteristics of the specific environment in which the biological agent is situated, such as the spatial configuration of the objects in the environment, as well as their relational properties. Each internal or external contextual element restricts behavior to some type of task appropriate to achieve specific goals useful to the well-being of the biological agent. In this sense, behavioral flexibility is modulated by the interaction of the agent-related and environmental context. Considering both contexts, agent-related and environmental context, biological agents autonomously set goals and select appropriate tasks to achieve them according to the situation, monitoring both their needs and motivations at the current moment as well as the possibilities of action that an specific environment offers them. Task selection would be, therefore, a function of these contexts.

Once a specific task has been selected, certain elements of the biological agent and the environment become relevant to achieve the task goal, these elements constitute the task-related context (Martin et al., 2012). This type of context overlaps with agent-related and environmental context only in those elements that allow biological agents to select the appropriate sensorimotor sequence to achieve the current selected task (Figure 1). These elements are essential to plan and execute goal-directed movements that dynamically change during task execution, such as the situated spatial body and object configuration (perceived *via* exteroception), the body posture of the biological agent (perceived *via* proprioception), and even the area around the biological agent in which objects can be grasped and manipulated, known as peripersonal space. Every time the biological agent moves its body or an object within the task space, the task-related context is constantly “updated" to consider these changes for the planning and execution of goal-directed actions. Since its nature is a function of the selected task, this context would be redefined every time the biological agent changes tasks. Thus, the dynamics of task-related context differ from agent-related and environmental context.

From a perspective that emphasizes embodiment for the development of cognition, behavioral flexibility is achieved when it is grounded in the constant monitoring of these three contexts (Figure 1). This monitoring occurs through signal processing of the interoceptive, proprioceptive, and exteroceptive sensory systems. Agent-related context processing is strongly linked to interoception and proprioception. Interoception allows the perception of physiological states of the body (Schulz, 2015), which play an essential role in determining appropriate tasks for survival. Proprioception informs about body posture, the changing body position during movement, velocity, and applied force (Tuthill and Azim, 2018). Since proprioception is essential for the planning of a task, it is also closely linked to the task-related context. On the other hand, exteroception allows the processing of environmental context. Through the visual, auditory, tactile, olfactory, and gustatory sensory modalities, exteroception captures information about the changes occurring in the environmental context (Wade, 2019). Processing environmental context helps to determine the task that better satisfies the biological agent's internal requirements according to the available external resources and the environmental configuration. Exteroception also provides information about the biological agent *situated* in the environment. Biological agents can obtain indirect information about their bodies with different exteroceptive sensory modalities, such as vision. This is essential to integrate information about the biological agent and the task being executed to guide action through perception (Kozak and Corneil, 2021). Therefore, exteroception is also associated with the task-related context.

In brief, the processing of the agent-related and environmental context provides flexibility in task selection and switching. Meanwhile, the task-related context processing provides flexibility in the planning of the sensorimotor sequence to perform a task and achieve the current goal under specific circumstances. Each contextual element constrains behavior driving the biological agent toward certain tasks and avoiding others. By endowing an artificial agent with the ability to process the current context, this agent would be able to select the task that is appropriate at a given time according to the specific circumstances at that moment. Most current artificial agents implementations, focus only on some contextual elements, those related to the task at hand, where mostly behavioral flexibility is not the object of study. The proposal here, is that, in order to achieve greater behavioral flexibility, contextual processing should be an important issue. That is why, here, some core contextual elements of each type of context have been identified that would allow artificial agents to modulate their behavior autonomously in a continuous cycle of context-sensitive actions. In Sections 3–5, we suggest core elements for the agent-related, environmental, and task-related context, respectively. At the same time, it will be addressed why these elements are considered core contextual elements for behavioral flexibility of biological agents and how they have been modeled in artificial agents.

## 3. Agent-Related Context

The agent-related context refers to elements associated with the physical and physiological structure of a biological agent that modulates behavior at different hierarchical levels of organization. At a higher level, this type of context plays a fundamental role for task selection. The agent-related context allows setting specific goals, which are a priority for the biological agent to stay alive during its coupling with the environment, restricting the set of appropriate tasks possibilities to satisfy an internal need or motivation. Physiological needs, intrinsic motivation, and emotions are elements of the agent-related context that have a strong impact on this level of behavioral organization. At a lower hierarchical level, the agent-related context plays a fundamental role in the planning and execution of goal-directed and reflexive actions. Once the agent has selected a task, aspects of the agent, such as body posture and peripersonal space become relevant contextual elements for the planning and execution of the specific task. Given their role in planning and executing tasks, these contextual elements fall within the overlap of agent-related and task-related context and will be addressed as elements of task-related context. In the following, physiological needs, intrinsic motivation, and emotions will be addressed. In the first part of each subsection, the reason why said element is considered a core element of the agent-related context in biological agents will be explained. Subsequently, the second part of each subsection will provide an overview of how the addressed contextual element has been modeled in artificial agents.

### 3.1. Physiological Needs

Physiological needs, such as hunger or sleep, are sensations evoked by internal states of the biological agent that indicate a lack of nutrients, energy, or any other of the many internal conditions necessary for survival (Taormina and Gao, 2013). When physiological needs are detected by the interoceptive modality, these must be regulated to maintain the homeostasis of the biological agent (Strigo and Craig, 2016). Physiological needs are associated with motivational states that constitute action drives related to survival (Maslow, 1958). For instance, when an animal is hungry, several types of hypothalamic neurons signal this need and drive a specific task, such as foraging (Schulkin and Sterling, 2019). Thus, physiological needs are core contextual elements that have a strong impact on behavior when they are detected (Ramirez-Pedraza and Ramos, 2021). Furthermore, they modulate task activation causing an effect on the relative desirability of different tasks. In the case of hunger, this averse sensation increases the desirability of foraging and decreases the attractiveness of other tasks not associated with getting food, such as playing (Loewenstein, 2011).

Like biological agents, artificial agents must have a baseline of certain states to function properly. For example, they must have a certain level of energy, integrity in their sensors, and maintain an optimum temperature for the proper operation of their motors. In artificial agents, to keep these internal states in optimal values, some studies have focused on modeling homeostatic systems (Stradner et al., 2009; Vargas et al., 2009; Yoshida, 2017; Man and Damasio, 2019; Kelkar, 2021). Generally, artificial agents must remain in a viability zone, the set of possible states in which the operation of the system is not compromised, allowing the activation of tasks that help to regulate those internal states when they exceed a predetermined limit.

Vargas et al. (2005) proposed a model based on an artificial neural network (ANN), and on a hormone production controller. Variations in external or internal states trigger the production of a specific hormone. The level of hormones alters internal states by driving neural networks' actions through stimulation of target neurons, affecting the input weights in the ANN to perform a certain task. Once the task has been accomplished, the hormone production controller receives a negative feedback signal that ceases the production of the hormone. In another study, Moioli et al. (2009) addressed the coordination of three coupled tasks in a mobile robot: exploring the environment while avoiding obstacles, searching for a light source when fatigue is high, and searching for a black stripe in the arena when the battery is low. They use three discrete-time artificial recurrent neural networks derived from a model inspired by gaseous modulators (Husbands et al., 1998). Each network is previously and separately evolved to accomplish a specific task. Subsequently, the output of the network is modulated by the levels of two simulated hormones associated with the levels of fatigue and hunger. The levels of hormones, together with an external stimulus, are responsible for determining the coherent coordination of behavior.

The homeostatic value of drives, together with the allostatic control for selecting appropriate behaviors to satisfy the intrinsic needs, have been modeled considering the relevance of the environmental context in Vouloutsi et al. (2013). Using a humanoid artificial agent, the designed Distributive Adaptive Control (DAC) architecture coordinated task selection depending on intrinsic drives during human-robot interaction. The DAC was based on reactive layers and adaptive layers. The reactive layers monitored the levels of the drives, sociality, exploration, survival, security, and play. The adaptive layers were responsible for the assignation of the drives' priorities, and behavior selection, depending on the current state of the world. The satisfaction of the drive and its associated homeostatic value controlled the expressed emotion of the system through facial expressions. In general, the DAC was capable of monitoring and satisfying artificial intrinsic drives, prioritizing them when several drives were competing, and organizing behavior depending on the perceived stimuli in a given environment. The DAC is a representative example of how modeling artificial internal drives and their homeostatic regulation allows an artificial agent to organize behavior autonomously responding to internal and environmental constraints.

In Kirtay et al. (2019), the authors implemented a model-free reinforcement learning (RL) framework to argue that emotion can be considered as an emergent phenomenon of a neurocomputational energy regulation mechanism in a decision-making task. This mechanism generates an internal reward signal to minimize the neural energy consumption of a sequence of actions. Each action triggers a process of visual memory recovery in which the actions to explore the environment are movements of the neck and the eyes to direct the gaze. According to the authors, the computational shortcut mechanisms on cognitive processes to facilitate energy economy give rise to emotions. In another work, Lewis and Cañamero (2016) study the role that pleasure plays in the selection of actions whether related or unrelated to the satisfaction of physiological needs. They evaluate the effects of different types of pleasures and show that pleasure, including pleasure not related to the satisfaction of physiological needs, has value for homeostatic management in terms of improved viability and greater flexibility in adaptive behavior.

A fundamental element for autonomy in artificial agents relates to energy. Most current artificial agents operate with batteries that must be replaced or recharged by the user (McFarland, 2009), so, self-charging robots would have a higher level of autonomy. In this regard, EcoBot-II is an interesting example designed to autonomously regulate its energy by converting unrefined insect biomass into useful energy using onboard microbial fuel cells with oxygen cathodes (Ieropoulos et al., 2005). The work described by Lowe et al. (2010) addresses energy-motivation autonomy where physiological information is generated by a simulated artificial metabolism as a microbial fuel cell batch. The grounding of behavior according to artificial metabolic constraints permitted the evolution of sensory anticipatory behavior in the form of simple pan/tilt active vision.

These studies show how physiological constraints impact not only sensorimotor activity but also emotional and motivational mechanisms. They allow the emergence of adaptive anticipatory behavior, prioritize tasks, and organize behavior according to the needs of artificial agents situated in a context. However, few studies address other physiological needs in artificial agents, such as engine integrity, or optimal operating temperature.

### 3.2. Emotions

There is no clear consensus about the definition of emotion, in part, because it can be defined based on its affective domain, as well as on its behavioral aspects that guide how biological agents act and respond to the environment (Soudry et al., 2011). It has been hypothesized that emotions evolved to drive behaviors that promote homeostatic processes, explaining why an emotional experience depends on the processing of interoceptive signals (Pace-Schott et al., 2019). For instance, physiological needs are strongly related to emotional experiences. Some basic emotions, such as fear, anger, disgust, sadness, happiness, and surprise could have been developed during the course of evolution and subserve adaptational strategies (Ekman, 1992, 2016).

Emotions can be generally defined as multifaceted, whole-body responses that involve coordinated changes in subjective experience, behavior, and peripheral physiology (Mauss et al., 2007). Emotions trigger responses from different biological systems, including facial expression, somatic muscle tone, tone of voice, and endocrine activity, to produce an optimal body milieu for an effective task response (Rolls, 2000). The role of these short-lived psychophysiological states encompasses coordinating behavioral response systems, shifting behavioral hierarchies, communication and social bonding, short-cut cognitive processing, facilitating storage, and recall of memories (Dolan, 2002; Phelps, 2006; Mulligan and Scherer, 2012; Tyng et al., 2017).

Emotions represent efficient modes of adaptation to changing internal and environmental demands, allowing behavioral flexibility or even triggering a task interruption when a sudden change occurs (Adolphs, 2016). They regulate behavior by associating the situation with states of positive or negative valence that express an appraisal involving a particular type of harm or benefit (Griffiths and Scarantino, 2001; Coifman and Bonanno, 2010). Thus, emotions are core contextual elements, providing direct agent-related information, regulating the selection of beneficial tasks, as well as the interruption of an ongoing task when necessary. Together, with physiological needs and intrinsic motivation, emotions drive biological agents toward behaviors that ensure their survival (Smith and Lazarus, 1990).

The computational modeling of emotions constitutes an area of growing interest in CR (Breazeal and Brooks, 2005; Ziemke and Lowe, 2009). The studies on emotions can be broadly divided into those that focus on their role in modulating behavior and those related to human-robot interaction (Arbib and Fellous, 2004). Here, we address models that highlight the role of emotions in the control of multi-task artificial agents (Kowalczuk and Czubenko, 2010; Ghayoumi and Bansal, 2016). In these approaches, artificial agents generally learn some predefined tasks and then find their high-level coordination. Some studies associate emotions with the expected utility of each behavior. From this perspective, emotions can be considered as triggers of behavioral action sequences according to some value. The higher the value, the higher the probability of a task to be selected.

Emotions have been modeled to drive RL algorithms (Moerland et al., 2017). Gadanho and Hallam (2001) proposed a model in which emotions provided a reward value and helped a mobile robot in determining the situations in which to reevaluate decisions. The robot must maintain its energy, avoid collisions and move around a closed maze-like environment. The addressed emotions were happiness, sadness, fear, and anger. The model was implemented using a recurrent neural network in which emotions influence the perception of the state of the world. In turn, this model was integrated into an RL architecture. The intensity of emotions is associated with the internal state of the artificial agent, determined by an energy deficiency and proximity to obstacles.

Marinier and Laird (2008) implemented a cognitive architecture called *state, operator, and result* (SOAR) (Newell et al., 1987; Laird et al., 2012) as a basis for the integration of an emotion module. Emotions allow the robot to assess what stimuli attend to (sudden, relevant, pleasant), and to decide what to do with the stimulus attended. Feelings serve as a reward signal for a four-wheel-driven mobile robot. Completing a task provides the robot with a positive reward. Daglarli et al. (2009) proposed a model in which emotions and a motivational system constitute the highest control level of the architecture. The motivation module assigns behavior gain coefficients which provide an increase or decrease of the impact of the behavior. In turn, emotions determine sequences of behaviors for the planning of long-term actions according to the probabilities of transition of the emotional and behavioral states. A hidden Markov model is implemented for behavioral and emotional transition processes.

Jitviriya et al. (2015) proposed a behavioral-emotional selection model based on a self-organizing map (SOM) and a discrete stochastic state-space Markov model. The artificial agent determines the most suitable behavior and emotional expression according to internal and external situations. Firstly, the artificial agent recognizes the external situation and determines its motivation. In turn, a cognition module is used for clustering the input stimuli (the intrinsic motivation and external situation) in a SOM. Then, the robot calculates the affective and behavioral factors. The behavioral-emotional selection system is implemented with a Markov model. The basic emotions simulated in this work are normal, hope, happiness, sadness, fear, and disgust.

Emotions have also been modeled using artificial evolution. Parisi and Petrosino (2010) suggested that adding an emotional circuit to the ANN that controls behavior leads to better motivational decisions and thus greater fitness. Artificial agents must eat and drink, eat and fly away from a predator, eat and find a mating partner, eat and care for their offspring, or eat and rest to recover from physical damage. Their results show that robots with ANN that include an emotional circuit behave more effectively than robots with ANN that do not. Other approaches that use ANNs for emotional modulation of tasks focus on increasing or decreasing the synaptic efficiency of specific populations of neurons associated with tasks (Belkaid et al., 2019). In general, artificial emotions have offered an elegant approach for behavioral flexibility in artificial agents, providing a unifying way to tackle different control issues.

### 3.3. Intrinsic Motivation

Intrinsic motivation (IM) could be defined as a natural desire or interest in carrying out specific behaviors just for the pleasure and satisfaction derived while performing them, rather than for external rewards or pressures (Ryan and Deci, 2000; Sansone and Harackiewicz, 2000; Oudeyer and Kaplan, 2008; Daddaoua et al., 2016). Exploration, manipulation, curiosity, and play are considered intrinsically motivated behaviors (Ryan and Deci, 2000; Reiss, 2004; Stagnitti, 2004). White (1959) called this psychophysiological need *effectance motivation* or *mastery*. The amount of effective interaction or degree of control biological agents can have on objects, tasks, themselves, and other agents naturally motivate behavior (Deci, 1975). IM allows biological agents to acquire knowledge about themselves and their world to effectively interact with the environment, being crucial for open-ended cognitive development and for autonomy (Deci, 1975; Perry et al., 2000).

It has been observed that the most motivating situations are those with an intermediate level of novelty, this is, situations between already familiar and completely new (Berlyne, 1960). When a biological agent performs a task, an emotion with a positive or negative valence is experienced as a result of how well or bad it is performing the task. Recently, it has been suggested that the monitoring of prediction error dynamics over time is a self-regulation mechanism behind IM (Schillaci et al., 2020b). Thus, a positive emotional experience is linked to a continuous decrease in prediction error, conversely, a negative emotional experience to a continuous increase in prediction error over time (O'Reilly, 2020; Schillaci et al., 2020b). This mechanism can help to explain how biological agents select their goals, as well as why behaviors such as being curious and playful should feel good (Kiverstein et al., 2019). IM involves an ongoing cycle of finding optimal goals and interesting tasks that evoke emotions with positive valence and it is, therefore, essential for learning and encouraging interaction with the environment (Gordon, 2020; Schillaci et al., 2020b).

The tendency to be intrinsically attracted to novelty has often been used as an example of IM for guiding exploration in artificial agents (Huang and Weng, 2002; Oudeyer et al., 2007). This approach is useful to acquire optimal information gain from the novel or interesting objects to create a more accurate model of the world through curious exploration based on an intrinsic reward inversely proportional to the predictability of the environment (Schmidhuber, 1991). In knowledge-based models, the *interestingness* of an action or event derives from the comparison between the predicted sensorimotor values, based on an internal forward model, and the actual values (Oudeyer and Kaplan, 2008). The intrinsic reward for each event is proportional to the prediction error of that event according to the learned model. Thus, interesting situations are detected by higher prediction errors.

IM allows artificial agents to autonomously select curiosity-driven goal-directed exploration behaviors and focus on goals with the optimal amount of reducible prediction errors (Schillaci et al., 2020b). Marsland et al. (2000) proposed a novelty filter using a SOM to learn representations of *normality* from sonar scans taken as a robot explores the environment. The features of the environment are clustered in the SOM. All neurons of the SOM are connected to a single output neuron. The connections to this output neuron represent the habituation process of biological neurons, recording the number of times that each winning neuron has fired. The output received from each winning neuron reduces with the number of times it fires. This allows the artificial agent to recognize novel or unusual features of the environment and forget features that repeat over time.

Competence-based models provide another measure of *interestingness*, given that it is the properties of the achievement process that will determine task selection (Oudeyer and Kaplan, 2008). Artificial agents pay little attention to those tasks that are already solved or unsolvable, for which the learning progress stays small (Colas et al., 2018). Thus, they engage in tasks associated with surprising or novel situations and can autonomously change tasks when their model has improved. The behavior is motivated by an intrinsic reward system that favors the development of competence rather than being directed to externally directed goals.

IM allows the progressive learning of more complex and hierarchically organized skills. Barto et al. (2004) proposed a strategy to explore the task space where each decision involves the execution of a temporally extended task. Agents are motivated to master tasks driven by the learning progress for each of them. Learning progress generates intrinsic rewards that determine action selection. Most implementations of IM use the RL computational framework given its inspiration in the brain reward systems (Eschmann, 2021). RL algorithms tackle the challenge of how an artificial agent can learn to approximate an optimal behavioral strategy, usually called a policy, while interacting directly with the environment. The optimality criterion of a problem is defining a reward function, an approximate solution is viewed as the skill of expertly controlling the given system (Sutton and Barto, 1998).

Luciw et al. (2011) proposed an artificial curiosity system based on RL for environmental exploration. The artificial agent builds an internal representation of its world through navigation. The reward signal is modified to contain two distinct components, one intrinsic and one external. The external component is the reward signal in classical RL, while the intrinsic reward signal is based on the measure of *interestingness* that is used as a motivational system to speed learning. The measure of *interestingness* assigns low values to patterns already known or that cannot be learned, and high values to patterns not known, but that can be discovered. The model assigns values for maximizing combined external and intrinsic rewards using a least-squares policy iteration with an internal forward model.

IM has focused on the exploration and manipulation of objects. Hart and Grupen (2012) propose that a single IM function for affordance discovery can guide long-term learning in artificial agents. Using RL, their function rewards the discovery of tasks such as finding, grasping, and placing simple objects. IM has been also used to improve the model of the artificial agent's body state and action space (Frank et al., 2014). This is achieved by guiding the exploration of states and actions using intrinsic rewards. Singh et al. (2010) consider an evolutionary perspective to define a new optimal reward framework that captures the pressure to design good primary reward functions that lead to evolutionary success across environments. They show that both intrinsic and extrinsic motivation can be understood as emergent properties of reward functions selected because they increase the fitness of learning of artificial agents across some distribution of environments. In general, IM allows learning to be more efficient by enabling the selection of novel tasks and goals with the optimal capacity for error reduction.

## 4. Environmental Context

Environmental context refers to the state of the environment surrounding a biological agent at a given moment, affecting how every sensory input is processed (Nikolić, 2010). It is related to the terrain characteristics, the climate, and illumination, as well as all the entities or objects in a scene (Bloisi et al., 2016). However, the arrangement of objects is a key factor in determining the environmental context. Each scene contains specific objects that appear with a certain probability, and the spatial relations among them also present regularities (Bar, 2004). Thus, the typical spatial configuration of the environment makes it possible to distinguish different types of environmental contexts. Environmental context restricts the tasks a biological agent can select at a given moment through the action possibilities that are provided in a situation. According to Gibson (2014), affordances refer to the possibilities for action that exist by virtue of the relational properties between the environment and an agent. From a cognitive robotics' view, affordances are acquired relations through bodily interactions of an artificial agent with its environment that provide support for planning, and reside inside the artificial agent as explicit relations that enable to perceive, learn, and act (Şahin et al., 2007).

Objects by themselves do not provide action possibilities, they need to be situated in a context to stand out as relevant, affording context-dependent interactions. Each environmental context offers a field of affordances to the biological agents according to the typical objects present in it Withagen et al. (2012) and Rietveld et al. (2018). Thus, the environmental context has a predictive impact on the behavior of the biological agent, by allowing certain actions to be taken, and restricting others. Furthermore, the situated body in the environment and object configuration have predictive power in the sensorimotor sequence necessary to interact with them. Attention is deployed to process the general configuration of the objects in the environment, prioritizing those relevant regions for bodily actions (Reed and Hartley, 2021). Together, these ideas are in line with the elements that have been suggested as necessary for physically grounding an affordance in an artificial agent. For doing so, it must be able to perform a behavior with an object given its morphology and its motor capabilities, must determine its relevance according to the artificial agent's intentions or goals, and must consider the spatio-temporal physical constraints of the objects in the environment to perform an action in the perceived context (Koppula and Saxena, 2014).

An embodied theory of spatial attention in a situated context is one that dynamically adjusts affordances of the body, the current environment, and the goals of the biological agent (Reed and Hartley, 2021). The spatial body and object configuration are fundamental elements of task-related context given their essential role in the planning and execution mechanisms for the selected task and will be addressed in Section 5. Even though many exteroceptive sensory modalities are used to obtaining environmental context information, for the sake of brevity, only visual information is addressed in this context, in both types of agents. Given the speed of contextual processing at the visual level, this sensory channel could be key to triggering predictions according to the context as stated by Bar and Aminoff (2003) and Bar (2007).

### 4.1. Spatial Configuration of the Environment

The semantic context of a scene might be extracted early enough to affect the perception of individual objects in it. Visual recognition of scenes is a fast, automatic, and reliable process (Oliva, 2005; Greene and Oliva, 2009; Lowe et al., 2018; Kaiser et al., 2019). Thorpe et al. (1996) have reported that complex natural scenes can be categorized under 150 ms. To explain this phenomenon, theories of visual perception have suggested a mode of processing based on specific spatial frequencies that would convey different information about the appearance of a stimulus (Kauffmann et al., 2015; Zhang and Li, 2019; Aghajari et al., 2020). High spatial frequencies (HSFs) represent abrupt spatial changes in visual information such as edges and correspond to configuration information and fine detail. Low spatial frequencies (LSFs) represent global information about the stimulus (Kauffmann et al., 2014). As stated by Bar and Aminoff (2003), a blurred partially analyzed image version of the visual input is projected rapidly from early visual areas toward the prefrontal cortex. LSFs in the image may provide coarse information of scenes and could reach high-order areas rapidly by conveying information through anatomical “shortcuts.” HSFs, then, convey fine details of the image more slowly (Kihara and Takeda, 2010; Kauffmann et al., 2017; Petras et al., 2019).

The blurred representation of environmental context activates expectations or predictions about the most likely interpretations of the input image in higher levels, which in turn is back-projected as an initial guess to the temporal cortex to be integrated with bottom-up processing (Bar, 2007). From this perspective, a correspondence between a novel input and an existing representation similar to the input stored in memory would be activated. Then, associated representations with that similar representation would be translated into predictions. Top-down processes may facilitate recognition by limiting the number of object representations that could be considered according to the experience of the biological agent (Bar, 2004). Environmental context representation is stored in unified memory structures called context frames. Some studies have suggested that associative representations integrate information about the identity of objects and their locations (Gronau et al., 2008). These structures would bring together information about the identity of objects that are most likely to appear in a specific scene, as well as about the probable spatial relations between these objects (Bar, 2004; Gronau et al., 2008). Brady et al. (2011) argue that individual items are not represented independently of other items on the same scene. Every scene could have multiple levels of structure, from the level of feature representations to individual items to the level of ensembles of objects. Each scene representation allows simulations regarding the activated context-specific category in support of situated action (Barsalou, 2020a).

Additionally, some studies have suggested that biological agents represent knowledge about where an object is typically used in conjunction with information about how the object is used. Peelen and Caramazza (2012) provided fMRI evidence that object representations in the anterior temporal lobes would convey information about where and how an object is typically used. This favors their structural coupling with the world, generating a field of affordances relevant to each environmental context. However, it is not entirely clear how these contextual associations are stored and integrated in the brain. Once biological agents learn regularities about this coupling, fast environmental context processing would allow them to generate predictions about possible interpretations of the situation, to simulate situations, and act according to what the environmental context dictates, selecting the appropriate task in each situation taking into account also the agent-related context.

CR usually model affordances as the relation between an action, a single object, and an action effect without explicitly considering other aspects of the environmental context in which objects are embedded. Some computational algorithms for learning affordances take into account an invariant environmental context implicitly (Yukie, 2011). From an embodied perspective, this restricts the interaction with the environment and the behavioral flexibility artificial agents can acquire during the learning process. However, there exist research on environmental context can be learned through behavioral experience in artificial agents during navigation. In their pioneering work, Nolfi and Tani (1999) proposed a hierarchical architecture of prediction networks that allows a mobile artificial agent to extract spatio-temporal regularities in a a simple and structured environment in order to infer its position, as well as to detect changes in the environmental topology. In their architecture, higher layers are trained to predict the next internal state of lower layers, extracting regularities at different levels of organization. The lower-level prediction layer extracts regularities such as “walls”, “corners” and “corridors”, while the higher-level prediction layer, by being exposed to higher-level internal states and to shorter sequences, extracts regularities which are hidden at the sensory level, such as ‘the left side wall of the large room' or “I am leaving the big room”. Each prediction layer is a feedforward network with recurrent connections. After being trained in an environment consisting of two rooms joined by a short corridor, the artificial agent is able to detect whether the corridor between the two rooms has been closed, whether a new obstacle has been placed in the environment, or whether the extension of one of the two rooms has been altered. This work is inspired by previous experiments described in Tani (1996).

In another study, Nolfi and Parisi (1996) implemented a genetic algorithm to simulate the evolution of a population of neural networks which control the behavior of mobile artificial agents that must explore efficiently an environment surrounded by walls (for a closer look at related studies see Nolfi and Floreano, 2004). In the experiments, artificial agents must be able to reach a circular target area in its environment that contains food. Since generations of artificial agents are not able to perceive the target area, they have to efficiently explore the environment to increase its chances of reaching the food arena without colliding with the walls. Each artificial agent is controlled by a feedforward neural network consisting of just an input and an output layer, without hidden units. The network includes a teaching subnetwork that determines how the standard network changes its connection weights during life. In this sense, the input generated by the teaching subnetwork can be influenced by the external context and it can teach different behaviors in different environments. Artificial agents are selected for reproduction according to their ability to explore one of the two possible environments, with dark or bright walls, respectively. Their results showed that individuals that are allowed to learn during their life perform better than those that do not learn. Although these types of studies are focused in learning environmental context through the agent's experience, these works usually pay less attention to the manipulation of objects.

On the other hand, there exist some studies that consider the environmental context to explore navigation and manipulation simultaneously (Sisbot et al., 2005). Mostly, these studies endow artificial agents with pre-set abilities so that they can perform various tasks in domestic environments. The knowledge of artificial agents usually includes databases of objects that they do not need to learn and the steps necessary to achieve goals are specified in advance. Blomqvist et al. (2020) presented a mobile manipulation system capable of perception, location, navigation, motor planning, and grasping. The artificial agent is mounted on an omnidirectional mobile base and can navigate using a 3D global pre-built map of his environment. The artificial agent builds an occupancy grid for navigation and locates itself in the environment by an online algorithm that estimates its position on the global map. During navigation, the artificial agent can detect objects through an RGB-based vision system, using a pre-trained ANN with a database of different objects. Once the task-related object is identified, the artificial agent extracts information about its position in space in order to grab it and the 3D geometry of the local scene is reconstructed in detail. Subsequently, grip pose detection algorithms are used to generate and classify a set of possible types of grasp. Finally, a path to the chosen grip position is planned and executed, the clamp is closed, and the object is retrieved from the table. The artificial agent can navigate in a laboratory, find an object on a table, take it and drop it in another place.

Asfour et al. (2006) implemented an architecture with a three-level hierarchical organization: task planning, synchronization and coordination, and execution level called sensor-actor level. Tasks are decomposed into subtasks that represent sequences of actions and contain the necessary information for execution, such as the parameters of the objects, and spatial information about the environment. The level of planning specifies the subtasks to achieve a goal and manages resources and skills. The coordination level activates actions sequentially or in parallel with the execution level. The execution level is based on control theory to execute specific control commands. This level uses specific local active models about the environment and objects. In the beginning, active models are initialized by global models, which integrate information from the environment, containing the database of objects, tasks, and abilities. The global model corresponds to long-term memory, while active models represent short-term memory.

Puigbo et al. (2015) endowed an artificial agent with predefined skills such as navigation, grasping, recognizing objects and people. They implemented the SOAR architecture as part of their approach (Newell et al., 1987; Laird et al., 2012). SOAR acts as the reasoner by selecting the actions that must be performed to achieve a goal. The control system is constituted by four main modules. Firstly, a vocal command is sent to the robot that is translated to text using an automatic speech recognition system. The semantic extractor module divides the received text into grammatical structures, from which the goal is generated. The goal is compiled in the reasoner module and sent as input to the SOAR cognitive architecture. The actions suggested by SOAR are translated as skill activations in the action nodes. The robot has information about the environment in five categories: (1) a map of the environment, (2) an ontology that contains all the actions, names of objects, people and places, (3) a database of 2D/3D models of objects that the artificial agent can recognize and grasp, (4) a database of faces that the robot can recognize and (5) a database with current knowledge of the state of the world, the artificial agent, objects and people. The information available allows the artificial agent to manipulate objects, navigate into a room, and interact with people.

Some efforts have been put into autonomous learning of the environmental context through the experience of artificial agents. However, these studies usually focus solely on environment navigation using mobile agents. Other studies have explored navigation and manipulation of objects at the same time. Generally, in these studies, environmental context is not acquired through autonomous learning. In some cases, artificial agents can plan sequences of actions. Nevertheless, the skills that they exhibit are not acquired through experience. However, it is clear that considering the environmental context extends the abilities that an artificial agent can exhibit.

## 5. Task-Related Context

Biological and artificial agents interact with objects through manipulation tasks, such as grasping or pushing. Each task involves a temporarily ordered sequence of sensorimotor states that leads to a specific goal (Grafton et al., 1998). To effectively plan and execute a sensorimotor task, agents need to acquire relevant information about themselves and the objects involved in the task. These relevant elements to achieve the task goal are determined once the task is selected and constitute the task-related context. The core elements for the planning and execution of a task suggested here are body posture, peripersonal space, and the situated body and object configuration (incoming sensory input) which dynamically change during task execution.

When grasping an object, information about its position and orientation is crucial to adapt the sensorimotor sequence accurately (Chen et al., 2014; Baltaretu et al., 2020). Given the spatial object configuration, it is possible to predict the sequence of actions that a biological agent will perform to achieve a specific goal. For instance, the type of grasp used to lift a glass would depend on whether the object is upside down or upright on a table (Rosenbaum et al., 2014). If the task involves two or more objects, the spatial relation between items becomes relevant to plan the task. Simultaneously, body posture is also essential for the execution of the sensorimotor task (Sarlegna and Sainburg, 2009). The sensorimotor sequence will also depend on the initial position of the body. This information can be directly acquired through proprioception or indirectly through incoming exteroceptive information, such as vision, which provides information about the configuration of the body situated within an environmental context.

Planning the sensorimotor sequence of a task implies that an agent has to predict the sensorimotor consequences product of its actions. During its execution, the prediction error, resulting from the difference between the predicted and the incoming sensory information, allows to dynamically adjust the sensorimotor sequence in accordance with the situated body and object configuration. Together, the body posture and object configuration would determine the sensorimotor sequence that would allow the agent to achieve the task goal (Rosenbaum et al., 2014). The body posture of an agent and its peripersonal space combined determine the location of a target relative to an extremity. The effective control of the body to avoid or manipulate objects requires an integrated neural representation of the body and the space around the body (Holmes and Spence, 2004).

### 5.1. Body Posture

Biological agents process information about the position of their limbs in space through sensory modalities, such as proprioception and vision (Sherrington, 1907; Grigg, 1994; Saunders and Knill, 2003; Saunders, 2004; Montell, 2019). The brain integrates this information in a multimodal neural representation known as body schema (Head and Holmes, 1911; Carruthers, 2008; Morasso et al., 2015; Hoffmann et al., 2020). The body schema allows to constantly monitor the body posture to trigger the planning and execution of goal-directed movements (Schillaci et al., 2016). When performing goal-directed movements, biological agents must integrate information about the body position and how this relates to extrinsic spatial coordinates of objects in the world (Sainburg et al., 2003).

Internal models have been suggested as the mechanism to code for body schema (Wolpert et al., 1995, 2001). These models allow biological agents to establish a causal relationship between their intentions and actions, as well as to anticipate the effects generated by their actions (Miall and Wolpert, 1996; Wolpert and Kawato, 1998; Kawato et al., 2003; Tanaka et al., 2020). Internal models integrate spatial body configuration and motor information to control movements and plan actions (McNamee and Wolpert, 2019). The body posture constitutes a core element of the task-related context given its determinant role in the planning and execution of action for a given task configuration (Zimmermann et al., 2012).

As infants do, artificial agents can also acquire a body schema. A common strategy is motor babbling (Demiris and Dearden, 2005; Kuniyoshi and Sangawa, 2006; Rolf et al., 2010; Houbre et al., 2021). During this process, artificial agents perform random movements which, in turn, cause changes in their sensory situation. These changes are then associated with the movements that cause them. Learning the spatio-temporal patterns that relate sensorimotor modalities with the body configuration allows artificial agents to distinguish between their own body and the environment (Diez-Valencia et al., 2019). In CR, internal models are a typical approach to allow artificial agents to acquire the sensorimotor representations necessary for prediction and action generation (Dearden and Demiris, 2005). Nevertheless, the computational tools to encode the spatial context of the body, the sensory situation, the movements as well as the approaches to map associations between them varies considerably (Schillaci et al., 2016; Nguyen et al., 2021). For example, Gama and Hoffmann (2019) study the acquisition of body schema in humanoid robots to construct map-like proprioceptive representations, resembling somatotopic representations within the brain. The joint angles of the robot are considered proprioceptive inputs and are obtained from different body configurations. Proprioceptive information serves as input to a modified SOM. The neuron activation in the maps encodes one specific joint or a combination between two or three of them as the receptive fields of neurons in the somatosensory cortex (Krubitzer et al., 2004).

Zhang et al. (2018) implemented an autoencoder to model proprioception in a humanoid robot. Interestingly, they do not consider joint angles directly as proprioceptive information, as it is typically done. Taking into account that the exact value of joint angles is unknown for biological agents, the joint configuration is the input to the network and the hidden layer is considered as proprioception. Using a multimodal variational autoencoder (VAE), Zambelli et al. (2020) proposed a system that enables an iCub to learn representations of its sensorimotor capabilities considering the spatial configuration of its body. The multimodal VAE is formed by multiple encoders and decoders, one for each sensory modality such as proprioception, vision, tactile, sound, and motor. In another study, Escobar-Juárez et al. (2016) endowed an artificial agent with the capacity of executing saccadic movements to focus a stimulus in the fovea as well as to carry out a hand-eye coordination task using multimodal representations. They proposed the Self-Organized Internal Models Architecture (SOIMA), a network of self-organized maps interconnected with Hebbian weights. SOIMA provides coupled inverse and forward models that allow multi-modal associations of sensory and motor information.

In these studies, body schema is not adaptable as has been reported in biological agents (lriki et al., 1996). Inspired by the flexibility of body representations, Nabeshima et al. (2006) proposed a biologically inspired model of body schema adaptation. The artificial agent reaches for and touches an object with its hand and learns to temporally integrate visual and tactile information in associative memory. If the recalled visual information is consistent with the currently obtained visual information, then the location of visual contact is considered as the location on the hand where the tactile sensation originated. If visual contact occurs not on the robot's hand, but on a given tool, then the robot is not able to adequately use the tool with the current hand trajectory controller, which induces the system to learn a new kinematic controller for the tool. In their model, the global memory is composed of two associative memories: a gating ANN to associate the visually detected target approach direction information with tactile information and, a non-monotone ANN associating tactile signals with the distance between the hand and the target. The authors suggest that tool use depends on the coherent unification of spatial and temporal aspects of multimodal information. Their model relies on the temporal integration of vision, touch and, proprioceptive information.

Learning algorithms are useful computational tools to create multimodal representations in CR, such as body schema (Hoffmann et al., 2010; Morasso and Mohan, 2021). From proprioceptive maps to multimodal representations, these studies endow artificial agents with the capacity to autonomously acquire contextual information about their own bodies. The most explored modalities in CR have been proprioception and vision. However, there is a growing interest in considering other modalities to provide artificial agents with greater behavioral flexibility (Dahiya et al., 2013; Zenha et al., 2018; Pugach et al., 2019).

### 5.2. Peripersonal Space

Peripersonal space can be understood as the reaching space of a biological agent, that is, the distance at which an object can be reached by the hand of the agent without moving the trunk (Cardinali et al., 2009; Serino, 2019). This region acts as an interface between the agent's body and the environment (Makin et al., 2008; Noel et al., 2021). Peripersonal space was also known as the flight zone and it would correspond to a margin of safety around the body (Dosey and Meisels, 1969). There is evidence about the involvement of peripersonal space in guiding involuntary defensive movements for protection. Some studies show that electrical stimulation of multimodal areas in the brain evokes a complex pattern of hand and arm movements in monkeys, similar to avoidance or defensive reactions, such as turning the head or raising the hand (Graziano et al., 2002).

Although biological agents perceive space as something continuous and unified, the processing of the peripersonal space is particularly characterized by a high degree of multi-sensory integration, mainly between visual and somatosensory (tactile and proprioceptive) information (Cardinali et al., 2009; Bertoni et al., 2020). The visually evoked responses of peripersonal multimodal neurons are modulated by the distance between the visual object and the tactile receptive field. In this way, visual information can be encoded with reference to the part of the body that contains the tactile receptive field (Cardinali et al., 2010). Such a map would give the location of the visual stimulus concerning the body surface in somatotopic coordinates. Additionally, peripersonal space includes different spatial representations, such as those around the hands and the face (Farne et al., 2005). Peripersonal space is crucial to guide movement (Graziano, 1999). It is a core contextual element of the task-related context given that it informs the body-related reachable spatial region where a specific task can be carried out.

Synthetic approaches have modeled peripersonal space centered on different parts of the body. Fuke et al. (2009) proposed a model that enables an artificial agent to acquire a head-centered peripersonal spatial representation using a SOM and Hebbian learning. Their model is inspired by the face representation in bimodal neurons found in the adjacent ventral intraparietal region of the brain, which codes the location of visual stimuli through the head-centered reference and connects visual and tactile sensations (Sereno and Huang, 2006). These neurons have been associated with the ability to avoid objects moving toward the face as a protective mechanism (Graziano and Cooke, 2006). Fuke et al. (2009) use proprioceptive information of the arm as a reference so that when the artificial agent moves his arm in front of his face the SOM is activated and learning occurs. Their simulated artificial agent learns the association of the visuo-spatial representation with the tactile representation of the face.

Juett and Kuipers (2019) recreate the learning process of peripersonal space in an artificial agent, by associating proprioceptive information of the arm and the visual perception of the hand and grippers of the agent. The peripersonal space is modeled using graphs. The nodes of the graph represent the state of the arm, and the edges correspond to safe movements. Paths represent safe trajectories from one pose to another. In their proposal, a reaching action emerges as a reliable way to hit and move an object in the environment. When an object is accidentally grasped, it moves dynamically with the hand, generating a grasping action. The learning process is modulated by a mechanism of IM and the artificial agent is capable of reaching and grasping objects based on unguided exploration.

Nguyen et al. (2019) modeled visuo-proprioceptive-tactile integration in a humanoid robot to develop reaching behaviors. They implemented a deep neural network that receives as input images from the cameras of the artificial agent and the position of the head, while the output is the arm position and tactile information of the hand and forearm. The network predicts arm configurations of successful reaching, together with information about the body part that would make contact with the objects. Finally, Jamone et al. (2012) endow an artificial agent with the ability to learn a representation of its own reachable space using motor experience. The reachable space map that they proposed uses a gaze-centered, eye-fixed reference frame. The position of a point in space can be encoded with the motor configuration of the head and eyes of the artificial agent. Their maps are implemented using a locally weighted projection regression ANN. After learning, the artificial agent is capable of estimating the reachability of a visually detected object, even before starting the reaching movement. Together with information about the configuration of the body, peripersonal space allows artificial agents to perceive the space that surrounds them in order to carry out processes of planning and executing manipulation tasks.

### 5.3. Situated Body and Object Configuration

During task execution, it is necessary for biological agents to continuously build a visual map of the current perceived spatial body position in relation to the spatial arrangement of objects. This exteroceptive information complements the perceived body posture *via* proprioception to guide and adjust sensorimotor sequences within the peripersonal space of the biological agent. Visual working memory and attentional mechanisms are coupled by means of the action that is being executed. An action plan guides the retrieval of the appropriate sensory memory representations, and when the expected outcomes of the action are successful the representations are robustly consolidated, leading to a more rapid retrieval in the future (Olivers and Roelfsema, 2020). Thus, the content of visual working memory is to serve future behavior, in such a way that action encoding occurs in response to those visual memories of relevant objects related to the anticipated actions (Boettcher et al., 2021).

A telling example is the execution of complex grasping actions (van Polanen and Davare, 2015). The spatial information of an object interacts with the information of its physical properties to control object-oriented hand movements. This spatial object configuration must be associated with information about the body configuration in order to map spatial information about objects into body coordinates (Colby, 1998; Graziano and Gross, 1998; Bertoni et al., 2020). Thus, the situated body and object configuration is a task-related contextual element that dynamically changes during the execution of the planned sensorimotor sequences. Action plans require working memory for anticipating and chaining multiple steps, as well as the use of attentional mechanisms that are guided by the situated recurrent feedback for learning appropriate sensory-action couplings (Olivers and Roelfsema, 2020). In case of not having vision or any specific modality, it would also be expected that an integration process be carried out with those modalities available to the agent to generate predictions according to its experience. Given that all the information for planning sensorimotor sequences can not be known in advance, selective attention to relevant information during the flow of action influences subsequent action plans (Reed and Hartley, 2021). The situated action cycle has particular outcomes that potentially change the agent-related and environmental context, and these changes can also trigger further iterations of the cycle (Barsalou, 2020b).

Many studies have taken the approach of “learning by doing” to explore the consequences of self-generated actions in artificial agents. Fitzpatrick et al. (2003) showed how robots learn the effect of pushing actions on objects. In each trial, the target was placed directly in front of the robot within the task space. Then, the artificial agent executed pushing actions from any of four different initial positions. During the task, two variables were monitored, the initial proprioceptive information of the hand position and at the moment of contact and, the direction of retinal displacement of the target. In another study, Hogman et al. (2016) endow a robotic system with the ability to learn different object categories in a pushing task. The authors define categories as action-effect relations or sensorimotor contingencies, modeling the effects in an object-centered representation. The pushing task was parameterized using position and velocity. The robotic platform learns the characteristics of translation and rotation of objects and acquires knowledge with a certain degree of confidence from repeated observations of action-effect pairs. The translation is computed as the Euclidean distance between the initial and the final positions and rotation is calculated through the dihedral angle between the two planes.

Other studies have focused on addressing tool affordances. In this case, learning corresponds to finding the mapping between a set of features that describe tools and the effects that these tools produce through actions on an object. Mar et al. (2018) propose an approach where a robot learns tool affordances through interaction and generalizes them for similar tools based on their 3D geometry. During the training phase, a set of drag actions is performed by an iCub with a large number of tools grasped in different pose orientations: right, front, or left. Each trial began by placing a tool in the robot's hand. After grasping the tool, the iCub automatically detects the tool-pose it was given. Once the tool was grasped and the robot's end-effector successfully extended to the tip of the tool-pose, the robot performed a series of exploratory actions to discover the tool-poses drag affordances. Tool affordances are learned as a regression between tool-pose features and action-effect vector projections using SOMs. In this study, the initial position of the objects that were dragged is constant and object-object relations between the tool and the target object are not considered. Tool affordances are also addressed in Nabeshima et al. (2006). Interestingly, this work discusses how manipulable objects, such as tools, can become incorporated into the agent's body schema through the temporal integration of multisensory information. The contribution of Nabeshima et al. (2006) is mentioned in Section 5.1, given the emphasis their research makes on the adaptation of body schema representation.

Understanding the effects of actions is essential for planning and executing robot tasks. Paus et al. (2020) show that predicting the effects of a pushing action enables goal-oriented manipulation tasks. In this research, an artificial agent learns internal models based on objects and the spatial relations between them. The perceived scenes are represented as object-centric graphs while the internal model predicts object pose changes due to the pushing actions. The object properties are stored in the nodes of the graph while edges contain relative spatial information between object pairs. The internal model is used to predict an output graph, from which the local object position, after the push, can be extracted. This study considers the initial and final position of objects explicitly in the model and also takes into consideration spatial relation between the objects in a scene.

Using previous knowledge is crucial for performing different tasks in new situations and contexts. Khazatsky et al. (2021) developed a situated controlled system for efficient self-supervised goal-conditioned RL. A robot was trained with several previous experiences of trajectories in different tasks and contexts and tested in new environments and tasks by sampling goals from a visuomotor affordance model. After training affordances (policies), the robot was tested in new environments which contained distractor objects as well as other objects that afforded an interaction, such as opening or closing a drawer or placing an object on a pot. Importantly, these objects that afforded an interaction were not previously seen but had similar characteristics related to what they afforded (e.g., drawer with a different type of handle). In this work, learning required generalization in terms of visual affordances and their associated behaviors during online interactions to collect more data and constantly improve the associated policy. As a consequence, the policy of grasping generalizes to grasping objects and the continual learning of new tasks is faster as it benefits from increasing prior knowledge. This method of visuomotor affordance learning allows online autonomous learning of tasks in new contexts, which highlights the relevance of using prior knowledge from other contexts and their related affordances for scalable and continuous learning.

In another study, QueiSSer et al. (2021) focused on the generalization of experiences in familiar task-related contexts to those in unfamiliar task-related contexts that can be achieved through learning during vision-based goal-directed planning. In their experiments, blocks of different colors were placed at random positions in the task space, and a robot arm with a video camera was required to stack them in an arbitrary configuration specified by a visual goal. The proposed model introduces a large network composed of dynamically interacting sub-modules, which incorporates a visual working memory sub-module (VWMs), a visual attention module, and an executive network for prediction of motor states and images. This network, also controls visual attention by masks visual images in the VWM. The large network is trained by using predictive coding. Additionally, an optimal visuo-motor plan to achieve a given goal state is inferred using active inference. The experiments showed that a process of generalization occurs due to the information processing developed through the synergistic interaction between the VWM and other modules during the course of learning, in which memorizing image contents and transforming them is dissociated. After learning, the performance of the model network in generating goal directed action plans using active inference was evaluated, in cases that involved manipulating blocks with novel colors. The results showed a significant improvement in performance when using an additional VWM, compared to a case using only a single VWM. The authors suggested that the essential aspect of the mechanism acquired through learning is dissociation of visual image contents from the mechanism for their manipulation. This proposed method allows the artificial agent to flexible adapt to the new characteristic of objects during goal-directed planning.

Affordances consider the change in the task space but the representation of this change can vary drastically during task execution and within contexts. An autonomous artificial agent must be sensible to contextual changes to be able to predict the best sensorimotor sequence when performing a situated task based on the most similar previously learned situations. The use of previous experience and affordance generalization is relevant when exploring new environments. However, here we want to highlight that task-selection in a given context is also guided by the current internal needs of an agent (agent-related context), as well as by the performance expectations the agent has associated with different tasks. In biological agents, these two elements are directly linked to emotional states.

## 6. Interactionist Model of Contexts

**Figure 2:**
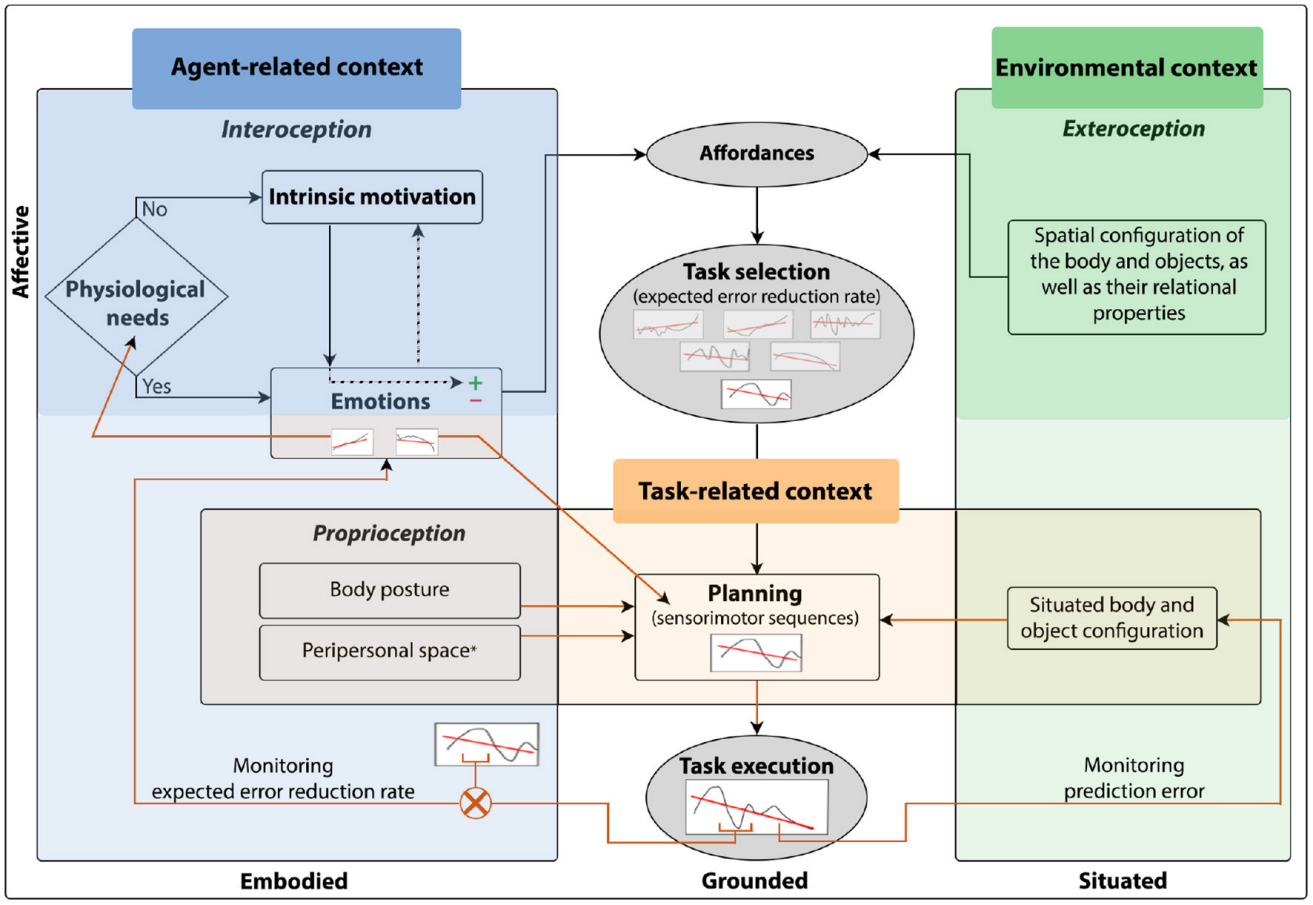
Interactionist model of contexts. Schematic representation of the three different types of context and the interaction of their core elements for selection, planning, execution, and when necessary switching of a task.

The interaction of agent-related, environmental, and task-related context for behavioral flexibility is analyzed in a schematic interaction model that integrates the core contextual elements (Figure 2), for task selection, its execution, and disengagement when necessary. In the model, each context is perceived by its main source of sensory information. For agent-related context, interoception and proprioception are key for providing an affective and embodied context. Exteroception is central for perceiving an environmental context in a situated manner, and finally, together, proprioception, interoception, and exteroception, are fundamental for grounding a task-related context during task execution. We suggest that the model presented here is a first approximation for grounding context in artificial agents. Artificial agents will be able to manage physiological needs, and intrinsic drives for learning, considering the situated perceived environmental factors. By means of perceiving the three types of contexts and their core contextual elements, artificial agents will behave according to the changing contextual conditions. This means that artificial agents will be more prone to become competent to autonomously select tasks that are of self-relevance to ‘survive', as well as tasks that promote learning, in a context-sensitive manner. This proposed interaction model is an idealized representation of the different contextual elements. In actual operation, as with other proposals (e.g., Barsalou, 2020b), one or more elements could be omitted, also, the sequence could be other than the one described here.

Biological agents learn regularities about the dynamics between the agent-related, environmental, and task-related context during their interaction with the world. It has been suggested that this association is encoded by different mechanisms, under the notion of internal models (Wolpert et al., 1995; Kawato et al., 2003; McNamee and Wolpert, 2019). Thus, biological agents learn to achieve their goals by anticipating the sensory consequences of their actions under specific contexts, and so, internal models are always context-dependent.

Internal models generate predictions about the most likely sensory consequences of self-generated actions. Biological agents always attempt to minimize the prediction error associated with predictions using two highly coupled strategies: by updating the internal model to generate better predictions or by fulfilling predictions through action to match the expected sensorimotor states (Friston et al., 2011; Clark, 2015). Furthermore, attention has been recently drawn to the importance of the monitoring of prediction error over time when executing a task. Thus, biological agents also learn the associated rate of how prediction error is being reduced while executing a task. This rate can be understood as changes in the velocity of prediction error reduction, in such a way that it informs how well or bad a biological agent is performing a task. This monitoring of prediction error dynamics and its associated reduction expected rate is thought to play a central role in emotions and well-being (Joffily and Coricelli, 2013; Van de Cruys, 2017; Kiverstein et al., 2019; Nave et al., 2020; Hesp et al., 2021).

The positive and negative valence experienced as we act is directly related to the success of the selected behavior in reducing prediction error at the expected rate. Additionally, due that prediction error dynamics are strongly related to emotions, it has been suggested that the monitoring of the rate of error reduction can be conceived as a self-regulation mechanism for guiding behavior in artificial agents (Schillaci et al., 2020b). Thus, an artificial agent can be intrinsically motivated to autonomously select a goal associated with an optimal reducible prediction error. The capability of monitoring the error rate reduction when performing the task, allows an artificial agent to autonomously ‘decide' if it should continue with the task when the pursued goal is being achieved, or if it has to be abandoned when no progress is achieved. In both scenarios, the artificial agent will be intrinsically motivated to select another goal that allows learning. It has been suggested that prediction error minimization is by itself rewarding. Decision-making based on rewards is replaced by the use of previous knowledge to avoid surprising states for survival, which is a sufficient condition to drive prediction error minimization (Friston et al., 2012).

In the model, physiological needs are central for determining which action has to be prioritized for maintaining the biological agent alive. When a physiological need is experienced, an associated emotion with a positive or negative valence, together with the environmental context, bring about the relevant affordances with which the biological agent can engage. As Rietveld et al. (2018) have suggested, biological agents respond to affordances in a context-sensitive way and affectivity is a central aspect of selective responsiveness to relevant affordances. To some extent, in the model, responding to relevant affordances for task selection and planning, can be understood as solicitations. Solicitations are those affordances that show up as relevant to a situated agent that feels immediately drawn to act a certain way (Dreyfus and Kelly, 2007). Responding with a preference to achieve a state of relative equilibrium and acting to correct for disequilibrium in relation to a dynamic field of multiple relevant affordances has been characterized as a tendency toward an optimal grip (Kiverstein et al., 2021). The best opportunities for improving the grip with the environment come from selecting those relevant affordances that are neither too complex, nor too simple, and can potentially lead to a desired outcome of equilibrium. Here, selecting the best task among solicitations is based on their associated expected error reduction rate. This rate is learned and constantly updated during situated action cycles, being directly linked to the current competence of the agent to achieve the desired outcome (for an implementation see Schillaci et al., 2020a).

When there are no physiological needs, intrinsic motivation brings the agent to explore its environment, eliciting positive emotions related to curiosity-driven behaviors. In this situation, task selection occurs in a similar fashion, the field of relevant affordances allows the agent to select the task best suited for exploration and learning, taking into consideration its expected error reduction rate. In this regard, inspiration comes from research, on infants, understanding preferences toward optimal exploratory behaviors. In general, infants prefer to attend to stimuli that evoke an intermediate rate of complexity (Kidd et al., 2012), and to those that contain unexpected patterns of data (Stahl and Feigenson, 2015) to be able to learn based on their current competences.

Thus far, all the above mentioned, refers to the upper part of the model, the shaded areas of both agent-related and environmental context. As an example, the functioning starts on the state of the physiological needs of the agent, is there a physiological need that must be fulfilled, when yes, this evokes and emotion and together with the element in the environmental context selects a tasks from the field of affordances to fulfill the respective need. When there is no physiological need to fulfill, then intrinsic motivation is the one driving the agent to select a task in the field of affordances. For both cases, the field of relevant affordances of a particular agent is dependent on its current concerns and competences, as well as the environmental situation, also, the optimal grip on the field of affordances dynamically changes as a result of this dependency (Bruineberg and Rietveld, 2014).

In the model, once the task has been selected, either for equilibrium maintenance and self-regulation or for exploration and manipulation of the environment, the task-related context emerges. First, for planning, the proprioceptive information, framed in the task related context (both overlapping with the two other contexts), becomes relevant for the planning of sensorimotor sequences. The selected sensorimotor sequence has an expected error reduction rate, schematically shown in the planning block of the diagram as an error occurring over time and its respective slope. Then comes the execution of the selected task. During execution of the task, two types of prediction error monitoring occurs in parallel. First, the monitoring of prediction error, the predicted sensorimotor consequences of actions are compared with the actual sensorimotor input for prediction error estimation. This is shown in the task execution block, again as error over time. The perceptual fast loop occurs as the situated body and object configuration changes as the execution of the task progresses, allowing corrections when necessary. This can be though of as the fast control loop of the execution of the task, involving internal models (depicted in the overlap yellow-blue, and the overlap yellow-green, respectively). Second, there is the monitoring of the expected error reduction rate. As the task is executed, the rate of error reduction in the monitored prediction error dynamics is compared with the expected error reduction rate. In other words, the accumulated prediction error over time when executing the task allows a direct comparison between the expected error reduction rate associated to the task and the actual prediction error dynamics.

The monitoring prediction error dynamics over time and its comparison with the expected error reduction rate signal how good or bad the agent is at performing the task, or how optimal is being its grip with the environment. This comparison is schematically shown in the comparator to the right of the task execution block. The minimization of prediction error and its relation with the expected reduction rate is thought to be at the core of emotions and valence of agents actions (Kiverstein et al., 2019; Hesp et al., 2021). When a faster than expected error reduction rate occurs, produces positive emotions, motivating the agent to continue with the task. A well-done feeling, also updates the expected error reduction rate for that particular task in that particular context. This is shown by the negative slope of the error at the lower left in the emotions block, with an arrow going back down to planning and execution. A rate of minimization of the actual error which is slower than the expected one can triggers a disengagement from the task. This difference will have a negative valence and bring the system back to the slow loop by means of monitoring its current physiological needs, as well as the other core agent-related and environmental contextual elements so as to select a different task. This might also occur when the agent is not capable to minimize the error. this is shown by the error at the lower right in the emotions block, with an arrow bringing the system back to monitoring of physiological needs. When the difference between the expected error reduction rate and the actual rate is not very large, the agent might continue with the execution of the task. Still, the comparison also has an emotional valence. A positive rate of reduction is an encouragement to continue as is, whereas a negative rate might be seen as a warning or as a signal for a necessary change in the manner the task is being planned and executed (Schillaci et al., 2020b).

The model shows two different temporalities in the rate that sensory changes occur. First, a low rate of sensory changes occurs while general properties of the contexts are processed to bring relevant affordances for task selection (intense blue agent-related context; intense green environmental context). This slow loop is represented in the model by black arrows interacting with the core contextual elements for task selection and planning. Second, when the task-related context emerges, a fast rate of sensory changes occur in the environment while executing the planned sensorimotor sequence of the task (light blue, green, and yellow). This fast loop is represented in the model by orange arrows interacting with the core contextual elements during task execution. In this regard, Marchi (2020) suggested that the line that distinguishes cognition and perception can be set by considering the functional levels of the processing hierarchy. Cognitive levels, the higher levels of the hierarchy, perform more abstract and general functions to represent general knowledge about contextual properties, and are not so susceptible to fast sensory changes that occur in the environment. On the contrary, perceptual levels, the lower levels of the hierarchy, are in close spatiotemporal proximity to sensory detectors, and are highly sensitive to fast sensory changes in the environment product of short-term actions (e.g., grasping, taking a step). Thus, the proposed model considers the sensitivity criterion proposed by Marchi (2020), in such a way that cognition is depicted by the slow loop for contextual information processing and task selection and planning, while perception is depicted by the fast loop, which is radically affected by fast sensory changes that occur during the task execution.

It is important to highlight the open question with regards to the optimal size of the time window in which prediction error dynamics has to be monitored. Different time windows of prediction error monitoring, starting from being very brief to relatively long, produce different patterns of emotional experience, as well as a different sensitivity to meaningful changes in the error reduction rate (Carver and Scheier, 1990). Recently, it has been suggested that the size of this time window should change dynamically according to ‘how well or bad things are going' with respect to the expected progress (Schillaci et al., 2020a,b). Thus, when the error rate constantly decreases, meaning the agent is doing well on the task execution, the need for error monitoring diminishes. On the contrary, if prediction errors are increasing, a more careful evaluation has to be done. In computational implementations, less monitoring implies the liberation of resources. In this regard, in the proposed model, the time window by which prediction error dynamics are monitored could change dynamically based on the experienced emotions product of the differences between the expected error reduction rate and the actual reduction rate. Additionally, here it is suggested that the time window can also be influenced by the level of familiarity of the perceived environmental context. When an agent becomes familiar with a particular context, the confidence or the precision related to relevant possibilities of action increases (Friston et al., 2017a,b). Thus, in a familiar environmental context, the tasks that tend to be selected are very likely to lead to preferred outcomes (pragmatic value), and as a consequence the expected rate of error reduction is very fast. In this scenario, previous experience guides the retrieval of robustly consolidated representations for action planning that will lead to the expected outcome (Olivers and Roelfsema, 2020). Given the pragmatic value of a selected task in a familiar context, the time window by which prediction error dynamics are monitored is decreased. On the contrary, in novel or unfamiliar environmental contexts the outcomes of a set of possible tasks tend to be uncertain. Accordingly, the tasks that can be selected in a novel environmental context tend to be for exploration and learning (epistemic value). Hence, their associated expected rate of error reduction is slow. As a consequence, the time window by which prediction error dynamics are monitored is increased until more experience is gained and appropriate sensory-action couplings are consolidated.

Finally, in line with Barsalou (2020b), the interactionist model of contexts presented here offers a grounded approach to perception, cognition, and behavior. The situated action cycles in the environmental context are grounded in the task that is being executed. Central to the model is the processing of physiological needs, as well as the constant monitoring of the prediction error dynamics, which are the base for emotional states. An optimal grip with the environment is provided by the equilibrium experienced by acting in a particular situation to reduce affective tension or disequilibrium (Rietveld, 2008). Thus, a situation improves by being responsive to those relevant affordances that potentially can bring about the experience of equilibrium. Further, the proposed model highlights the particular role of the different sensory systems such as interoception, proprioception and exteroception in cognitive processes associated with the modulation of behavior. From this perspective, cognitive and perceptual processes not only occur in the brain, but are distributed in the dynamic coupling, full of affectivity, between the brain, the body, and the environment. Thus, the interactionist model of contexts is then: a) embodied in the processing of the physiological needs of agents, their morphology and their sensorimotor capabilities, b) affective, as agents act to improve the context-sensitive grip on a dynamic field of relevant affordances, c) situated in the environmental context, the current body and object configuration that, together, make the relevant affordances stand out for task selection and planning, and finally, d) grounded in the situated action cycles during task execution that trigger the processing of fast multimodal sensory changes, as well as the two types of prediction error monitoring that occurs in parallel.

## 7. Discussion

Context processing plays an essential role in autonomy and behavioral flexibility of biological and artificial agents. Essentially, context is involved in all cognitive, perceptual and behavioral aspects. Endowing artificial agents with the ability to process the context in which they are situated would allow them to prioritize goals and tasks that are important for their internal self-regulation and to promote their learning and mastery of the environment. This makes context and its processing a key element for CR. The vast majority of studies in CR consider one or more contextual elements, however, the concept of context is rarely explicitly addressed. There is consensus that context acts as a set of restrictions that influence behavior, but, the discussion is open on what the notion of context actually is. Given the relevance of context not only in behavioral autonomy and flexibility but in cognition in general, this work aims to motivate the discussion about context processing within CR. In this paper, context is treated as encompassing all those elements of the agent and the environment that have an impact on decision-making and behavior. The essence of context is complex given the diverse nature of its components. Here, to address global context, a distinction has been made, analyzing context as agent-related, environmental, and task-related context. The agent-related context is characterized by elements such as physiological needs, emotions, intrinsic motivation, as well as the morphological aspects of the body. The environmental context relates to the characteristics of the specific environment in which the agent is situated, such as the spatial configuration of the objects in the environment, as well as their relational properties. Finally, the task-related context is characterized by elements that dynamically change during the execution of the task, such as the situated spatial body and object configuration (perceived *via* exteroception), the body posture of the agent (perceived *via* proprioception), and its peripersonal space. It is suggested here, that the three types of context must be monitored at all times. When an agent is involved in the execution of a task, most of its attentional resources are devoted to achieving the goal. However, an agent can not afford to stop monitoring its physiological needs or its surroundings, big changes in any context must be attended in order to guarantee survival.

For each type of context, their core elements are analyzed separately, and several implementations in CR, representative for each core element, are described. Generally, each study focuses on different cognitive processes using a variety of mathematical and computational tools for their implementations. Here, it is proposed that establishing agent-related, environmental and task-related context allows a rapid identification of the elements considered in each study, regardless of the process modeled or computational tool used. In this sense, the classification of implementations made here, according to the core contextual elements, can shed light about the scope and limitations of the study of context in CR. At the same time, further research can be framed using this classification as a guide toward more autonomous and flexible behavior in artificial agents.

The main aim of this work is to explore and understand how the three contexts and their core elements should interact to provide behavioral flexibility in biological and artificial agents. A model is proposed integrating these core contextual elements considering their interactions and different temporalities during task selection and execution. The model gives great importance to the role of monitoring prediction error dynamics, as well as the expected error reduction rate. The agent-related context, together with the environmental context bring about a field of affordances at a given moment. Task selection is made on the field of relevant affordances according to the expected prediction error reduction rate for each task. Monitoring of prediction error dynamics allows online corrections of the planned sensorimotor sequence, by comparing predictions with incoming sensory information. All these, occur in the grounded task-related context during the agent's situated action cycles. Monitoring prediction error over time, as the task is executed, and comparing it with the expected prediction error reduction rate allows an agent to be sensible to its performance. This sensitivity signals if it is appropriate to continue execution, when results are positive and it “feels good,” or autonomously switch task, when things occur not as expected, and the task becomes “frustrating.” The model also includes two temporal resolutions, a slower one for cognition and a faster one for perception and situated action cycles.

Finally, the interactionist model of contexts suggested here is embodied, affective, and situated, by means of the monitoring of the agent-related and environmental core contextual elements. Additionally, it is grounded in the processing of the task-related context and the associated situated action cycles during task execution. The model suggests how artificial agents should monitor the core contextual elements of the agent-related and environmental context to give rise to the task-related context based on the field of relevant affordances, their associated expected error reduction rate and its positive or negative emotional valence, reflecting a tendency toward an optimal grip. This capability allows agents to autonomously select a task, its planning, execution, and monitoring for behavioral flexibility. In this regard, the model could shed light on the complexity of the dynamics of affordances' activation and to what extent the context filters this activation (see Borghi, 2018, for an extensive analysis of this issue). The modeling of context is essential to study the structural coupling between agents and their environment. The model presented here aims to contribute in this direction, as well as in clarifying the notion of context for behavioral flexibility, not only in artificial agents, also in biological agents.

## Data Availability Statement

The original contributions presented in the study are included in the article/supplementary material, further inquiries can be directed to the corresponding author.

## Author Contributions

DV contributed to the conception of the study and drafted the first manuscript. AC, GS, and BL guided and contributed to the writing of the article. All authors contributed to the conceptual design of the work and to the development of the proposed model. Finally, all authors contributed to manuscript revision and approved the submitted version.

## Funding

This research was supported by the Consejo Nacional de Ciencia y Tecnología (CONACyT; grant no. 517333).

## Conflict of Interest

The authors declare that the research was conducted in the absence of any commercial or financial relationships that could be construed as a potential conflict of interest.

## Publisher's Note

All claims expressed in this article are solely those of the authors and do not necessarily represent those of their affiliated organizations, or those of the publisher, the editors and the reviewers. Any product that may be evaluated in this article, or claim that may be made by its manufacturer, is not guaranteed or endorsed by the publisher.
